# Supplementary material for: Iterative improvement in the automatic modular design of robot swarms
Source: PeerJ Comput Sci. 2020 Dec 7;6:e322. doi: 10.7717/peerj-cs.322 (PMC7924708; doi:10.7717/peerj-cs.322)
Supplement: Supplemental Information 3 [file peerj-cs-06-322-s003.zip › argos3/doc/api/standalone/a00519_source.html]

ARGoS: plugins/robots/generic/simulator/colored\_blob\_omnidirectional\_camera\_rotzonly\_sensor.h Source File


- Main Page
- Related Pages
- Namespaces
- Classes
- Files

- File List
- File Members

# plugins/robots/generic/simulator/colored\_blob\_omnidirectional\_camera\_rotzonly\_sensor.h

Go to the documentation of this file.

```
00001 #ifndef COLORED_BLOB_OMNIDIRECTIONAL_CAMERA_ROTZONLY_SENSOR_H
00002 #define COLORED_BLOB_OMNIDIRECTIONAL_CAMERA_ROTZONLY_SENSOR_H
00003 
00004 namespace argos {
00005    class CColoredBlobOmnidirectionalCameraRotZOnlySensor;
00006    class COmnidirectionalCameraEquippedEntity;
00007    class CLEDEntity;
00008    class CControllableEntity;
00009    class COmnidirectionalCameraLEDCheckOperation;
00010 }
00011 
00012 #include <argos3/core/utility/math/rng.h>
00013 #include <argos3/core/simulator/space/space.h>
00014 #include <argos3/core/simulator/sensor.h>
00015 #include <argos3/plugins/robots/generic/control_interface/ci_colored_blob_omnidirectional_camera_sensor.h>
00016 
00017 namespace argos {
00018 
00019   class CColoredBlobOmnidirectionalCameraRotZOnlySensor : public CCI_ColoredBlobOmnidirectionalCameraSensor,
00020                                                           public CSimulatedSensor {
00021 
00022    public:
00023 
00024       CColoredBlobOmnidirectionalCameraRotZOnlySensor();
00025 
00026       virtual ~CColoredBlobOmnidirectionalCameraRotZOnlySensor();
00027 
00028       virtual void SetRobot(CComposableEntity& c_entity);
00029 
00030       virtual void Init(TConfigurationNode& t_tree);
00031 
00032       virtual void Update();
00033 
00034       virtual void Reset();
00035 
00036       virtual void Destroy();
00037 
00038       virtual void Enable();
00039       
00040       virtual void Disable();
00041                         
00042    protected:
00043 
00044       bool                                     m_bEnabled;
00045       COmnidirectionalCameraEquippedEntity*    m_pcOmnicamEntity;
00046       CControllableEntity*                     m_pcControllableEntity;
00047       CEmbodiedEntity*                         m_pcEmbodiedEntity;
00048       CPositionalIndex<CLEDEntity>*            m_pcLEDIndex;
00049       CPositionalIndex<CEmbodiedEntity>*       m_pcEmbodiedIndex;
00050       COmnidirectionalCameraLEDCheckOperation* m_pcOperation;
00051       bool                                     m_bShowRays;
00052 
00053    };
00054 }
00055 
00056 #endif
```

---

Generated on 10 Jul 2018 for ARGoS by 
 1.6.1
